# Conformational Plasticity in Amyloid Assemblies: A Paradigm Shift from Structural Rigidity to Functional Adaptability

**DOI:** 10.1021/acscentsci.5c00983

**Published:** 2025-06-06

**Authors:** Alan H. Weible, Xiaoguang Wang

**Affiliations:** † William G. Lowrie Department of Chemical and Biomolecular Engineering, 2647 The Ohio State University, Columbus, Ohio 43210, United States; ‡ Sustainability Institute, The Ohio State University, Columbus, Ohio 43210, United States

## Abstract

The divergence of conformational ensembles of hIAPP in response to different types of mutations and PTMs has been decoded by leveraging the scanning tunneling microscopy-based probability interpretation technique.

## Graphical abstract

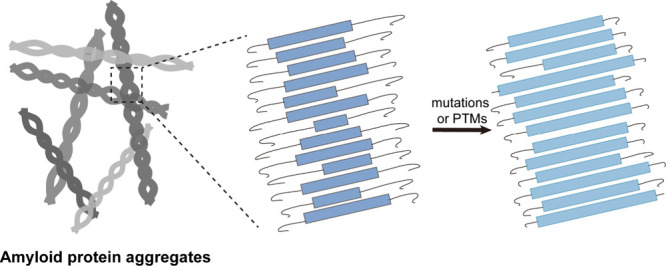

The classical paradigm in structural biology, rooted in the “one sequence, one structure, one function” dogma, has long portrayed proteins as static entities adopting singular, thermodynamically stable conformations to execute defined biological roles.^1^ This view, largely shaped by early X-ray crystallography studies, emphasizes structural rigidity as a prerequisite for functional specificity. For instance, classical models consider amyloid aggregates as rigid, highly ordered β-sheet assemblies, with subunit conformations within a given β-sheet considered identical (Figure 1). However, the debut of advanced techniques like nuclear magnetic resonance (NMR) and cryo-electron microscopy (cryo-EM) has unveiled a hidden dimension of protein behavior—conformational heterogeneity.^2–3^

**Figure 1:**
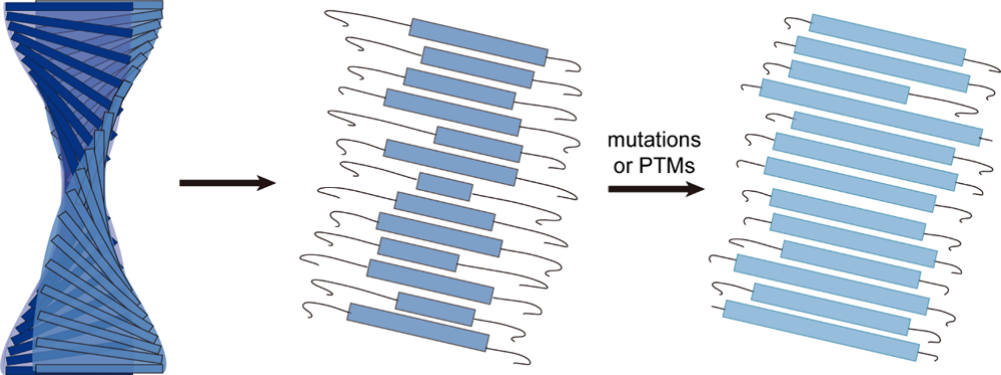
Classical models depict β-sheet assemblies as rigid, highly ordered supramolecular architectures (left). In contrast, STM studies uncover a dynamic conformational ensemble of metastable substates coexisting within β-sheet assemblies (middle). These conformational landscapes are structurally reprogrammed by mutations and PTMs, highlighting their inherent plasticity (right).

Emerging studies are demonstrating that proteins inherently accommodate diverse conformational states, forming dynamic ensembles that enable functional plasticity.

Mutations and post-translational modifications (PTMs) amplify this diversity, acting as molecular perturbations that reshape energy landscapes and exponentially expand the structural and functional repertoires encoded within these ensembles.^4^ This paradigm shift casts conformational plasticity not as a biological anomaly but as a fundamental driver of protein evolution and adaptability.

Yet, a critical gap remains: How do mutations and PTMs dynamically remodel conformational landscapes, and how do these changes translate into functional divergence?

In this issue of *ACS Central Science*, C. Wang, M. Wang, L. Yu, and co-workers report the successful use of the scanning tunneling microscopy (STM)-based probability interpretation (SIPI) technique to probe the conformational ensemble of human islet amyloid polypeptide (hIAPP) assemblies and the divergence of conformational plasticity in response to different types of mutations and PTMs in the primary structure of hIAPP.

hIAPP, misfolding into β-sheet-rich aggregates, is linked to type 2 diabetes. The authors show that wild-type hIAPP adopts 17 coexisting conformational substates interconnected by 60 distinct interstrand interaction modes, forming a dynamic “conformational ecosystem”.^5^ Their findings challenge the traditional view of amyloids as rigid, ordered fibrils. To probe how genetic and post-translational variations modulate this system, the team analyzed four hIAPP variants: the disease-associated mutant (hIAPP S20G), the terminus-substituted mutant (hIAPP COOH), the multiple-site mutant (rIAPP R18H), and the artificial mutant carrying PTM (hIAPP S20p).

The results are striking in their bidirectional complexity.^5^ While the S20G mutation and COOH terminus substitution reduce conformational diversity (to 15 and 12 substates, respectively), phosphorylation at S20 expands the ensemble to 22 substates. This shift extends to interstrand interaction networks: the rIAPP R18H mutant increases interaction types by 60% (to 97), whereas S20p nearly doubles them to 113. Such enriched plasticity and more complex intermolecular interactions might correlate with altered aggregation kinetics. These observations position sequence variations as molecular “dials” that fine-tune both structural diversity and functional outputs, defying the notion of mutations as mere disruptors of native folds. The authors also report the remodeling effect of mutations and PTMs on the energetic landscapes of inter-β-strand interactions within the β-sheets formed by hIAPP. Four types of variants are observed to profoundly remodel the topography, the weighted average energy, and the root-mean-square roughness of the energy funnel adopted by the hIAPP β-sheet. In contrast to classical models, STM studies reveal that β-sheet assemblies comprise a dynamic conformational ensemble of metastable substates. These conformational landscapes are remodeled by mutations and PTMs, underscoring their inherent plasticity (Figure 1).

By establishing a predictive “sequence–conformational ensemble–property” framework, the authors reveal that β-sheet assemblies generate structural diversity 2 orders of magnitude greater than sequence variation alone.^5^ This dramatic expansion arises from combinatorial interplay: each of the >10 substates per variant engages in ∼10 distinct interactions, creating vast interaction repertoires.

Such inherent disorder transforms amyloid aggregates into evolvable platforms, where natural selection acts not on fixed structures but on dynamic interaction networks.

This mechanistic insight bridges amyloid pathology and molecular evolution; the plasticity enabling pathogenic strain diversity in Alzheimer’s and prion diseases may drive functional innovation in natural protein systems.

Wang et al.’s work offers transformative applications. The STM imaging-based probability interpretation (SIPI) methodology provides a roadmap for engineering adaptive biomaterials. By rationally tuning conformational ensembles, rather than targeting one single low-energy state, researchers can design peptide assemblies with protein-like functionalities.

For instance, ensembles mimicking allosteric regulation could yield smart materials responsive to pH, temperature, or ligand binding. In therapeutics, targeting conformational landscapes rather than monomeric structures may combat amyloidosis more effectively; small molecules stabilizing nonpathogenic substates could halt toxic aggregation. Moreover, the study recontextualizes amyloid “disorder” as a latent functional reservoir. Although β-sheet chaos underlies neurodegeneration, it also encodes untapped potential for catalysis, molecular sensing, and evolvable nanodevices. This duality mirrors the role of genetic mutations as drivers of both disease and innovation.

This research represents a paradigm shift in structural biology, dismantling the artificial dichotomy between order and disorder.^5^ Their relevant work reveals that the conformational ensembles as well as the interpeptide interactions of hIAPP are found to evolve with time in the growth and plateau phases of aggregation.^6^ By redefining amyloid aggregates as sophisticated, evolvable systems, their work illuminates a path toward harnessing conformational plasticity for biomedical and technological breakthroughs. As we move beyond the static “structure–function” dogma, the next frontier lies in decoding the language of conformational landscapes, presumably a universal grammar governing protein behavior from ancient evolutionary innovations to modern disease mechanisms. This study not only answers long-standing questions but also frames new ones: How do cells exploit conformational chaos to regulate signaling networks? Can we design synthetic ensembles with evolutionary capacity? The answers, much like the proteins themselves, will emerge from dynamic exploration of nature’s most versatile molecular ecosystems.
